# Impact of hearing rehabilitation programs on presbycusis management: a systematic review and meta-analysis of randomized controlled trials

**DOI:** 10.3389/fragi.2024.1299964

**Published:** 2024-10-21

**Authors:** Zhanhang Zheng, Shuhong Qin, Ruilin Li, Wenjuan Wang, Chenxingzi Wu

**Affiliations:** Faculty of Nursing, Guangxi University of Chinese Medicine, Nanning, Guangxi, China

**Keywords:** age-related hearing loss, hearing aid efficacy, hearing rehabilitation program, meta-analysis, old adult

## Abstract

**Background:**

In the field of audiology, numerous studies have sought to understand and improve hearing rehabilitation programs for older adults afflicted with presbycusis. Despite this, the field lacks uniform standards pertaining to the intervention methods, frequency, and duration of such programs. These discrepancies have led to varying test results and inconsistent findings across multiple studies.

**Objective:**

This meta-analysis aimed to evaluate the efficacy of hearing rehabilitation programs in enhancing the utilization of hearing aids among older adults with presbycusis.

**Methods:**

We conducted a comprehensive exploration of PubMed, Embase, Cochrane Library, and Web of Science to identify randomized controlled trials assessing the role of hearing rehabilitation programs for patients with age-related hearing loss. The search period spanned from the inception of each database to September 12, 2024. Outcomes were synthesized using RevMan 5.4 software.

**Results:**

Eight studies met the inclusion criteria, involving 598 patients (290 in the intervention group and 308 in the control group). It was observed that hearing rehabilitation programs significantly diminished self-perceived hearing impairment [MD = −5.80, 95% CI = (−8.16, −3.44), *p* < 0.00001] and negative emotional states [MD = −1.66, 95% CI = (−3.02, −0.29), *p* = 0.02], while enhancing hearing aid utilization [MD = 0.22, 95% CI = (0.08, 0.36), *p* = 0.002]. Nonetheless, these programs did not significantly augment patients’ satisfaction with their hearing aids [MD = 0.09, 95% CI = (−0.17, 0.26), *p* = 0.66].

**Conclusion:**

Hearing rehabilitation programs significantly improve hearing aid outcomes, reduce self-perceived hearing impairment, and alleviate negative emotional states in patients. However, the current body of evidence is insufficient to conclusively indicate that these programs enhance patient satisfaction with daily hearing aid usage.

## Introduction

Presbycusis, or age-related hearing loss, is a leading sensorineural deficit among the elderly population. Approximately 66% of individuals over the age of 70 are estimated to have some degree of presbycusis, however, only 20% of these individuals are estimated to receive appropriate therapeutic intervention (Goman and Lin, 2016; Fulop et al., 2019). The most common clinical manifestation is bilateral symmetrical sensorineural hearing loss, which has been widely documented in the literature (Gates and Mills, 2005; Agrawal et al., 2008; Bowl and Dawson, 2019). This condition significantly impairs elderly individuals’ social interaction, mental health, self-care ability, and cognitive function, and can potentially progress to cognitive decline and even dementia (Jafari et al., 2019; Zhang et al., 2021).

Primary interventional strategies for presbycusis encompass both hearing aid prescription and cochlear implantation (Briggs, 2019). In light of their cost-effectiveness, convenience, and non-invasiveness, hearing aids are typically the preferred modality for managing mild to moderate age-related hearing loss (Sprinzl and Riechelmann, 2010). However, their efficacy can be hindered by the elderly population’s limited understanding and acceptance of these devices. Consequently, the need for a comprehensive hearing rehabilitation program is of crucial importance. This type of program aids patients in adapting to hearing aids, ensures the devices are functioning optimally, augments the effectiveness of hearing rehabilitation, and, ultimately, enhances patient satisfaction (Lu et al., 2019).

Hearing rehabilitation programs typically integrate health education and patient follow-ups, with the effectiveness of health education evaluated through monitoring patients’ hearing aid usage. However, a review of the existing literature presents a complex picture due to variations in study design, sample size, and outcome measures. Notably, an earlier study reported encouraging results with telephone-based consultations as part of the rehabilitation strategy, while a more recent study, which utilized physician-led, in-person consultations, yielded less optimistic outcomes (Lundberg et al., 2011; Zhao et al., 2022). This discrepancy in findings underscores the current knowledge gap about the optimal strategies for hearing rehabilitation. To address this, our study proposes a meta-analytic approach to better understand the potential impact of hearing rehabilitation programs on the efficacy of hearing aids among presbycusis patients. This approach is intended to provide a comprehensive evaluation of how these programs may enhance hearing aid outcomes in patients with age-related hearing loss.

## Methods

### Search strategy and study selection

The Preferred Reporting Items for Systematic Reviews and Meta-Analyses (PRISMA) guidelines were used for the review design and methodology (Page et al., 2021). Search strategy and study selection In this systematic review and meta-analysis, we evaluated randomized controlled trials to determine the effectiveness of hearing rehabilitation program interventions on hearing aid usage among older adults with hearing impairment. The search was limited to English-language publications. We conducted a systematic search of databases including PubMed, EMBASE, Web of Science, and Cochrane Library up to September 12, 2024. The search used a combination of keywords such as “age-related hearing loss” and “Aural Rehabilitation”, refined by terms such as “clinical trial” and “older adult: aged >60 years”. We also manually reviewed reference lists of the identified studies to include any relevant additional studies. This study was registered on PROSPERO(CRD42024560809).

In line with the PICOS (Participant, Intervention, Control, Outcome, Study Design) framework, we included randomized controlled trials (RCTs) that studied adults primarily aged ≥60 years with age-related hearing loss who were hearing aid users. Studies that included a small proportion of participants younger than 60 years were considered if the majority of participants were older adults. The included trials had to implement hearing rehabilitation programs, which could include components such as health education, lectures, or expert consultations, delivered either online or offline. The control group received standard care. We excluded observational studies, meta-analyses, letters to the editor, conference papers, republished literature, animal studies, and studies where the primary population was under 60 years of age. Additionally, we excluded trials where hearing loss was caused by non-age-related factors, such as trauma or congenital conditions, or where participants were not hearing aid users.

### Outcome

We leveraged several assessment tools to measure the outcomes. The Hearing Handicap Inventory for the Elderly (HHIE) (Newman and Weinstein, 1988) served as a self-reported gauge for hearing handicap, where increased scores pointed towards more severe activity limitations and participation restrictions. To assess the benefits of hearing aids, we employed the International Outcome Inventory for Hearing Aids (IOI-HA) (Cox et al., 2000), with superior outcomes denoted by higher scores. We utilized the Satisfaction with Amplification in Daily Life (SADL) (Cox and Alexander, 1999) to capture both patient satisfaction and perceived benefits of hearing aids. Meanwhile, the severity of anxiety and depression symptoms was measured using the Hospital Anxiety and Depression Scale (HADS) (Zigmond and Snaith, 1983), with higher scores correlating with more pronounced symptoms.

### Data extraction and quality assessment

Two reviewers independently assessed all articles and extracted data, with any disagreements resolved by a third party upon reviewing the original article until consensus was achieved. The reviewers independently screened titles and abstracts, with a full-text review conducted when abstracts did not provide sufficient information pertaining to the inclusion criteria. A standardized data extraction form was used to capture details such as the first author’s name, publication year, sample size, mean age, intervention duration, and outcome measures. Study quality was evaluated using the Cochrane Systematic Review Manual’s bias risk assessment tool (Egger et al., 1997; Ahmed et al., 2012), with evaluation indicators including randomization, allocation concealment, blinding of participants and personnel, blinding of outcome assessment, completeness of outcome data, selective reporting, and other sources of bias.

### Statistical analysis

All analyses were conducted using RevMan 5.4 software (provided by the Cochrane Collaboration). Heterogeneity testing and calculation of 95% confidence intervals (CIs) were performed. Mean differences (MDs) and standard deviations (SDs) of measurement data were used as indices of systematic evaluation, expressed via 95% CIs. Heterogeneity across included trials was assessed using I^2^ and Q statistics, with significant heterogeneity defined as I^2^ > 50.0% or *p* < 0.10. In the presence of assumed heterogeneity, a random-effects model was used to combine effect sizes following the exclusion of significant heterogeneity. If clinical heterogeneity was significant, a source of heterogeneity was analyzed, or a subgroup analysis was conducted. If the heterogeneity was too substantial to be resolved, a descriptive analysis was conducted. Statistical significance was set at *p* < 0.05.

## Results

### Literature search and study selection

**FIGURE 1:**
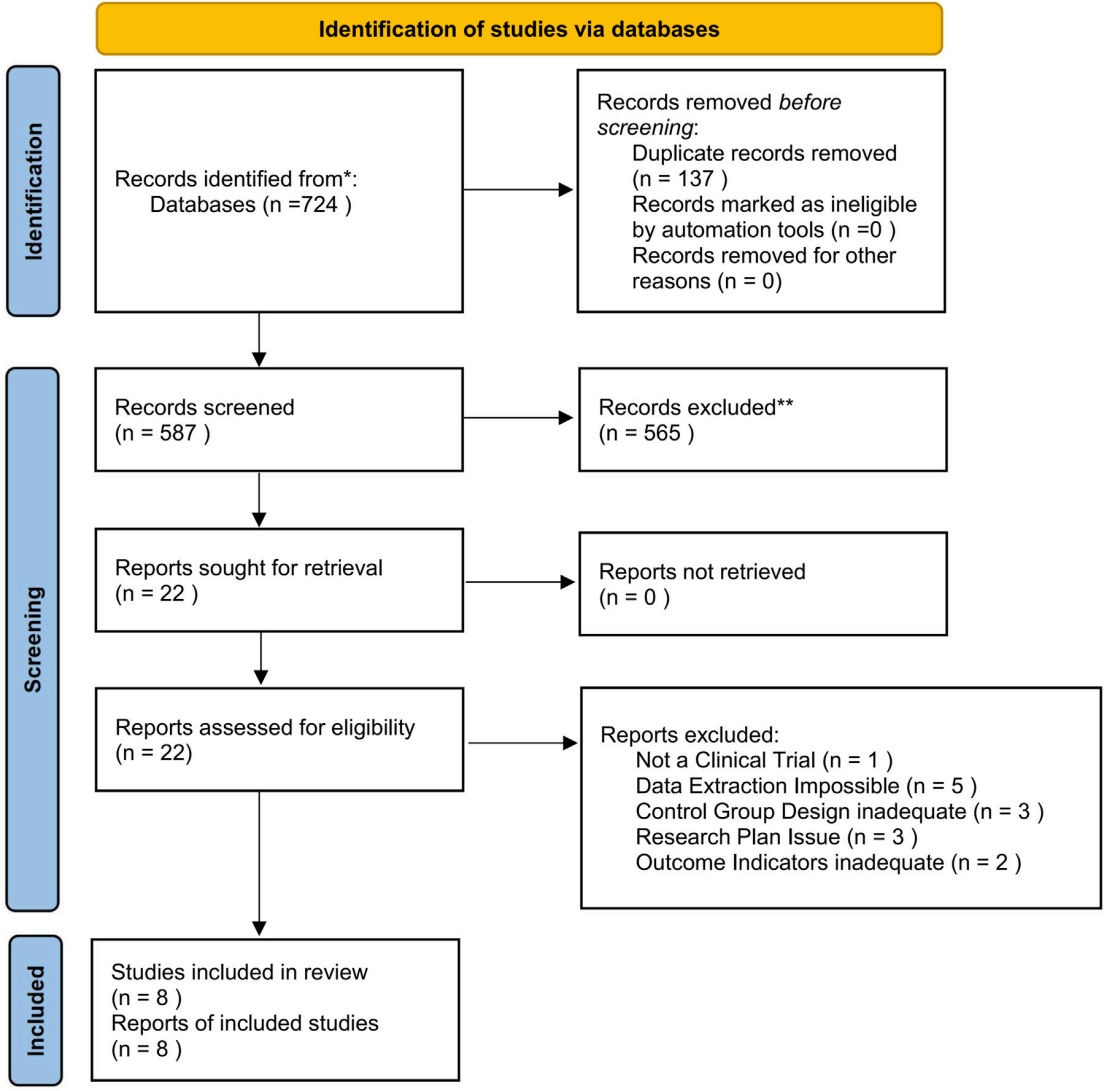
Flow diagram of study screening and selection procedure.

The initial database search yielded a total of 724 articles. After eliminating 137 duplicates, 22 potential studies remained for consideration. Following a thorough review of the titles and abstracts, eight full-text articles met the predefined eligibility criteria and were included in the analysis. Figure 1 delineates the process of the literature selection.

### Quality assessment of included studies

All eight studies included in the analysis reported the use of random grouping and explained their method for random allocation. Allocation concealment was mentioned in five studies, and a blinding methodology was used in three studies. The integrity of the results, selective reporting of research findings, and other potential sources of bias were assessed as low risk.

### Study characteristics

The final analysis incorporated eight studies involving 598 participants, with 290 in the intervention group and 308 in the control group. The fundamental characteristics of the included studies are summarized in Table 1.

**TABLE 1:** Basic characteristics of included studies (n = 8).

Included studies	Country	Follow-up method	Participants		Intervention		Test time	Follow-up duration	Age	Dropout rate	Definition of hearing loss	Duration of hearing aid use	Intervention frequency	Rehabilitation program design
			Intervention group	Control group	Intervention group	Control group								
Elisabet2011	Swedish	Internet	25	26	Rehabilitative online education vs. Internet discussion group	Weekly forum discussion	Upon study completion; 6-month follow-up	5 weeks	Mean 63.5 years	12.35%	HHIE score ≥ 20 (indicative of some hearing problems)	> 1 year	Weekly	Reading, weekly homework
Elisabet2014	Swedish	Internet	38	38	Online rehabilitative intervention	Supplied reading materials	Upon study completion; 3-month follow-up	5 weeks	Mean 69.3 years	15.78%	HHIE score ≥ 20 (indicative of some hearing problems)	> 1 year	Weekly	Reading, home training, interaction with audiologist, peer interaction through online forums
Han2022	Korea		18	19	hearing rehabilitation therapy	Standard treatment	4-month follow-up, 8-month follow-up	2 months	Mean 73.9 years	7.50%	Moderate-to-severe SNHL (PTA 41–80 dB across 500–4,000 Hz)	Intervention group: Mean 3.75 years, Control group: Mean 3.25 years	Weekly	Face-to-face interviews, training sessions, daily homework for self-rehabilitation
Hilde2015	Netherlands Belgium	Home visit	63	65	Dual sensory loss protocol	Routine hearing rehabilitation.	3-month follow-up	3 months	Mean 81.5 years	13.28%	Reported hearing disability	38.6% of patients: 0–5 years, 61.4% of patients: >5 years	3–5 times per week	Hearing aid usage, environmental adjustments, hearing assistive devices, communication strategies
Lundberg2011	Swedish	Telephone	33	36	Telephone-based educational program	Supplied reading materials	Upon study completion	5 weeks	Mean 68 years	7.59%	Conductive or mild-to-moderate SNHL (20–60 dB HL across 500–2000 Hz)	> 1 year	Weekly	Weekly home assignments, telephone consultations
Malmberg2017	Swedish	Internet	20	21	Internet-based aural rehabilitation program	Supplied reading materials	Upon study completion; 6-month follow-up	6 months	Mean 71.1 years	12.10%	Conductive or sensorineural hearing loss (20–60 dB HL across 500–2000 Hz)	> 3 months	Weekly	Reading, home training, interaction with audiologist, peer interaction via internet-based forums
Malmberg2023	Swedish	Internet	51	52	Clinical online hearing support	Standard treatment	Upon study completion	5 weeks	25% < 65 years, 75% > 65 years	22.79%	HHIE score ≥ 20 (indicative of some hearing problems)	> 3 months	Weekly	Weekly reading guides, submission of assignments
Zhao2022	Canada	Interview and internet	42	51	Physician Consultation	Regular follow-ups	Post-3 months hearing aid fitting	3 months	Mean 70.9 years	15.70%	SNHL > 25 dB (measured at 0.5, 1, 2, 4 kHz or 2, 3, 4 kHz)	Newly fitted	Once during the study	Physician consultation

### Efficacy of hearing aids

A total of 8 studies (Lundberg et al., 2011; Thorén et al., 2011; Thorén et al., 2014; Vreeken et al., 2015; Malmberg et al., 2017; Han et al., 2022; Zhao et al., 2022; Malmberg et al., 2023), involving 598 participants (290 in the intervention group and 308 in the control group), were included in this analysis. The results of the meta-analysis indicated that hearing rehabilitation programs significantly reduced self-perceived hearing handicap [MD = −5.80, 95% CI = (−8.16, −3.44), *p* < 0.00001] and negative emotions [MD = −1.66, 95% CI = (−3.02, −0.29), *p* = 0.02], while also enhancing hearing aid usage among elderly patients with hearing loss [MD = 0.22, 95% CI = (0.06, 0.36), *p* = 0.002]. However, no significant improvement was observed in terms of satisfaction with amplification [MD = 0.09,95% CI = (−0.17,0.26), *p* = 0.66]. All comparisons underwent heterogeneity testing. No significant heterogeneity was found in the comparisons.

**FIGURE 2:**
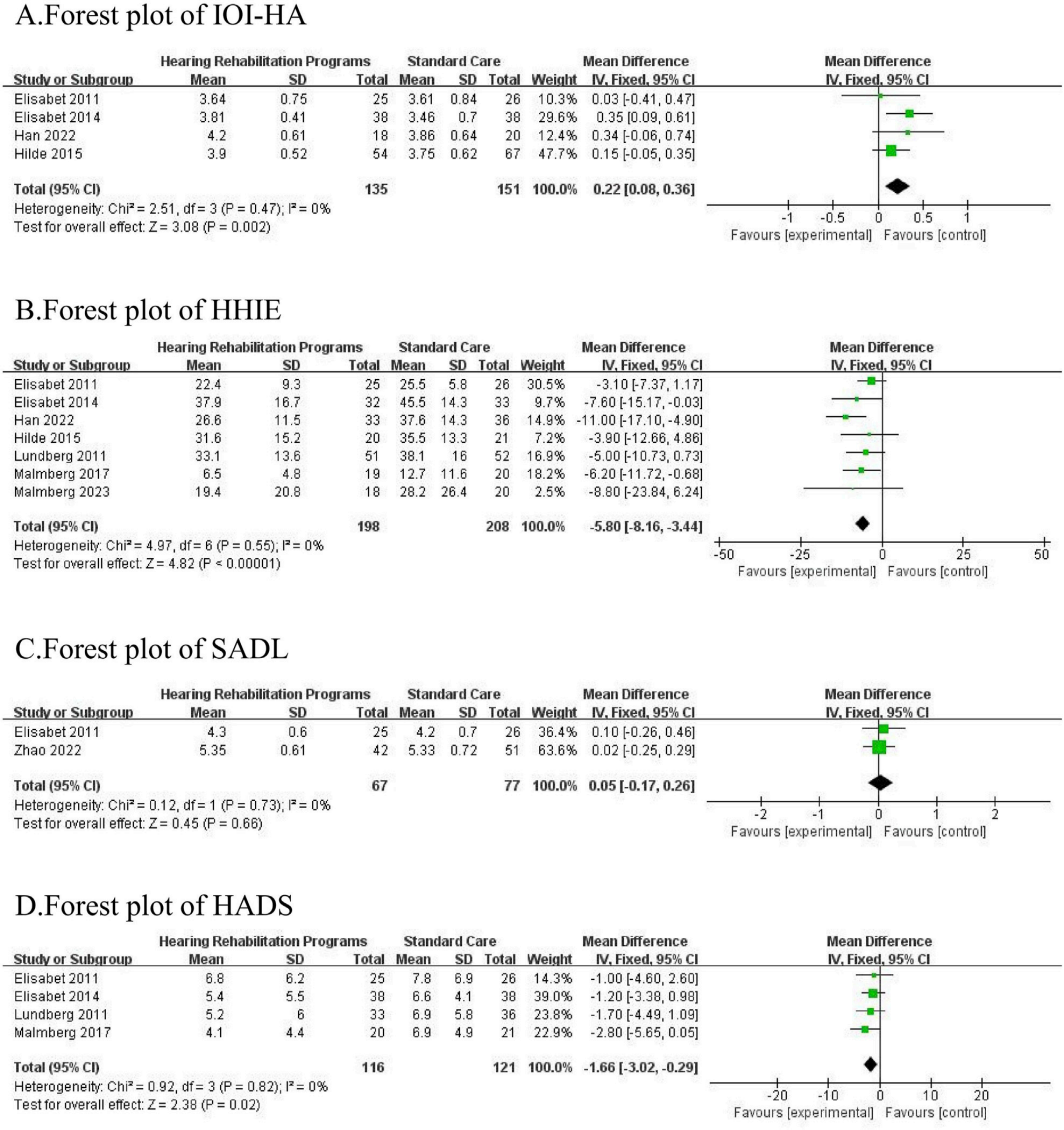
**(A)** Forest plot of IOI-HA. **(B)** forest plot of HHH. **(C)** forest plot of SADL. **(D)** forest plot of HADS.

Figure 2 Forest plot and meta-analysis of the included studies. (Figure 2A represents the synthesis of four studies that analyzed the impact of auditory rehabilitation programs on the effectiveness of hearing aid usage, utilizing the IOI-HA as the primary evaluation tool. Figure 2B displays the combined findings of seven studies that examined the influence of auditory rehabilitation programs on self-reported hearing impairment, assessed by the HHIE. Figure 2C presents the comparative results of two studies that investigated the role of auditory rehabilitation programs in determining hearing aid satisfaction, employing the SADL as the metric of measurement. Figure 2D offers a comparison drawn from four studies that focused on the implications of auditory rehabilitation programs on negative emotional states, evaluated through the HADS. The meta-analysis reveals a statistically significant enhancement in hearing aid efficacy, a reduction in self-reported hearing impairment, and an alleviation in negative emotions due to auditory rehabilitation programs (*p* < 0.05). However, these programs do not significantly improve hearing aid satisfaction (*p* > 0.05). IOI-HA: International Outcome Inventory for Hearing Aids, HHIE: Hearing Handicap Inventory for the Elderly, SADL: Satisfaction with Amplification in Daily Life, HADS: Hospital Anxiety and Depression Scale.)

## Discussion

This investigation was primarily designed to evaluate the influence of hearing rehabilitation programs on the usage of hearing aids among individuals suffering from presbycusis. Our meta-analysis incorporated eight studies with a total of 593 participants. It was observed that hearing rehabilitation programs could enhance patients’ understanding of hearing aids and promote a harmonious interaction between patients and their hearing aids. This enhancement was achieved through the development of tailored educational plans and consistent follow-ups, thereby amplifying the effectiveness of hearing aid usage. Nonetheless, due to variances in the design and intervention periods of current hearing rehabilitation programs, results varied across different studies. This research identified that the efficacy of a hearing rehabilitation program heavily relies on the follow-up method and duration of the intervention. Different follow-up approaches, such as internet-based or telephone-based methods, show varying degrees of effectiveness. Elderly individuals, in particular, may experience greater difficulties with internet-based methods due to lower acceptance of technology, leading to higher dropout rates (Hoerger, 2010; Li et al., 2024). In contrast, telephone-based follow-ups offer more immediate and interactive engagement, which is essential for addressing patient concerns and improving adherence to hearing aid usage. Therefore, understanding how design elements such as follow-up methods, duration, and frequency affect the outcomes of hearing rehabilitation programs is vital for optimizing patient care and enhancing the long-term use of hearing aids.

### Impact of age and other sensory impairments on hearing rehabilitation outcomes

Different age groups may respond differently to hearing rehabilitation interventions. Studies such as those by Malmberg et al. (2023, 2017) included participants under the age of 60, which may influence the generalizability of the findings to older populations. Younger participants may have greater adaptability to internet-based follow-up methods and demonstrate better adherence due to their familiarity with technology. In contrast, older adults might benefit more from traditional methods like telephone-based follow-ups, which offer direct and immediate communication. Therefore, interventions should be tailored to accommodate the specific needs of different age groups to maximize effectiveness.

Moreover, the presence of other sensory impairments, such as visual or tactile deficits, can significantly alter the efficacy of hearing rehabilitation programs. For instance, patients with visual impairments may find it challenging to engage with written or screen-based content, a challenge noted by Vreeken et al. (2015). Tailored approaches, such as audio-based materials or more personalized interventions, could help address these challenges. Considering additional sensory impairments during the design of hearing rehabilitation programs is critical to ensuring that all patients, regardless of their sensory limitations, can fully benefit from these interventions.

### Interactive and intuitive follow-up methods in hearing rehabilitation programs

As communication technology evolves, the landscape of follow-up methods in hearing rehabilitation is changing. While Internet-based follow-ups are becoming increasingly common, traditional telephone-based follow-ups continue to play a crucial role, particularly for elderly patients. Studies indicate that older adults often show a lower acceptance rate of Internet usage, which can contribute to higher drop-out rates in online follow-up programs (Swanepoel de et al., 2010). This challenge underscores the importance of selecting a follow-up method that aligns with the needs and preferences of this demographic. Telephone follow-ups provide immediate and interactive engagement, allowing audiologists to address patients’ questions and concerns more effectively (Wang et al., 2024; Zhang et al., 2024). This direct communication not only fosters a better understanding of the rehabilitation process but also enhances patients’ confidence in managing their hearing aids. Furthermore, the instant interaction afforded by telephone communication can help mitigate feelings of anxiety and depression related to hearing loss (Lundberg et al., 2011). However, it is essential to recognize the potential benefits of Internet-based follow-ups as well. With advancements in technology, online courses and video telecommunication are emerging as promising alternatives (Humes et al., 2019; Han et al., 2024). These methods can deliver information in a more intuitive and engaging manner, which may enhance understanding and retention among elderly patients. By incorporating video content and interactive elements, healthcare providers can create a more enriching rehabilitation experience. Ultimately, a balanced approach that considers both traditional and modern follow-up methods may be the most effective strategy. Tailoring follow-up interventions to accommodate the unique needs of older adults will likely lead to improved outcomes in hearing rehabilitation, ensuring that patients receive the support necessary to enhance their quality of life.

### Intervention duration and cycles of hearing rehabilitation programs

Currently, the intervention duration of hearing rehabilitation programs ranges from several weeks to months (Humes et al., 2014). From the two studies reporting patients’ satisfaction with hearing aid usage, we cannot conclusively demonstrate the effectiveness of hearing rehabilitation programs in improving this satisfaction. From the perspective of intervention time, due to the low acceptance of new knowledge among the elderly, only 3–5 weeks of intervention is not enough to enable elderly patients to fully accept the intervention content. Furthermore, the study by Zhao et al. (2022) had a dropout rate exceeding 15%, which undoubtedly influenced the test results. These factors contribute to some discrepancies in the outcomes. This study found that periodic interventions in the hearing rehabilitation program are required to sustain the effects. In the study by Thorén et al.(2011,2014), patients’ subjective hearing impairment and anxiety and depression symptoms increased 3 months post-intervention. This suggests that in implementing the hearing plan, researchers should focus on maintaining the rehabilitation effects. We propose that two to three interventions, each lasting for 4–5 weeks within 1 year, is a worthwhile approach. This is consistent with a previous systematic review (Michaud and Duchesne, 2017). Continuous and regular interventions would allow patients to supplement forgotten knowledge timely and maintain the intervention effect.

### Limitations

Despite our best efforts, this study still bears certain unavoidable limitations. Firstly, the interventions in the included studies varied significantly, not only in terms of duration but also in the timing of outcome assessments. These discrepancies contribute to a certain degree of heterogeneity, which may affect the comparability of results across studies. Secondly, several studies included in the analysis had small sample sizes, which could compromise the statistical power and generalizability of the findings. Additionally, the lack of uniformity in defining key outcome measures may further obscure the interpretation of results. Furthermore, potential biases, such as publication bias or selective reporting, could also impact the validity of our conclusions. Future research should focus on conducting high-quality, multicenter, and large-sample randomized controlled trials to address these limitations and provide more robust evidence for the efficacy of hearing rehabilitation programs. It is essential to standardize intervention protocols and outcome measures to enhance comparability and reliability in future meta-analyses.

## Conclusion

Hearing rehabilitation programs improve outcomes for hearing aid users with presbycusis, but their efficacy is influenced by various factors, including follow-up methods, intervention duration, and the design of the rehabilitation programs, such as educational content, weekly tasks, and awareness-building activities. A comprehensive approach, which considers both the structure of the intervention and patient-specific factors, such as age and comfort with technology, enhances effectiveness. Personalized, timely follow-ups via internet or phone improve engagement, while regular, repeated interventions reinforce knowledge and support long-term outcomes, especially addressing knowledge attrition.

## Data Availability

The original contributions presented in the study are included in the article/supplementary material, further inquiries can be directed to the corresponding author.
